# Simultaneous bridging and antegrade stent-in-stent placement via endoscopic ultrasound-guided hepaticogastrostomy using novel multi-hole metal stents

**DOI:** 10.1055/a-2776-5537

**Published:** 2026-01-22

**Authors:** Haruo Miwa, Hiromi Tsuchiya, Shotaro Tsunoda, Kazuki Endo, Ritsuko Oishi, Yuichi Suzuki, Shin Maeda

**Affiliations:** 1 26437 Gastroenterological Center, Yokohama City University Medical Center, Yokohama, Japan; 2 Department of Gastroenterology, Yokohama City University Graduate School of Medicine, Yokohama, Japan

**Fig. 1:**
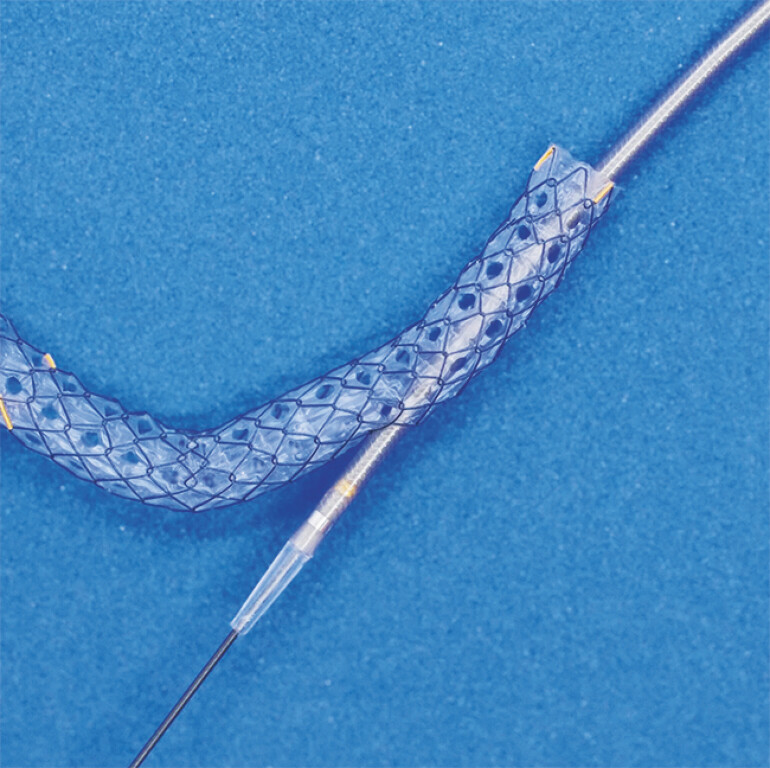
HANAROSTENT Biliary Multi Hole Benefit (M.I. Tech Co., Ltd, Pyeongtaek, South Korea). The 5.9-Fr slim delivery system can pass smoothly through the side hole.

**Fig. 2:**
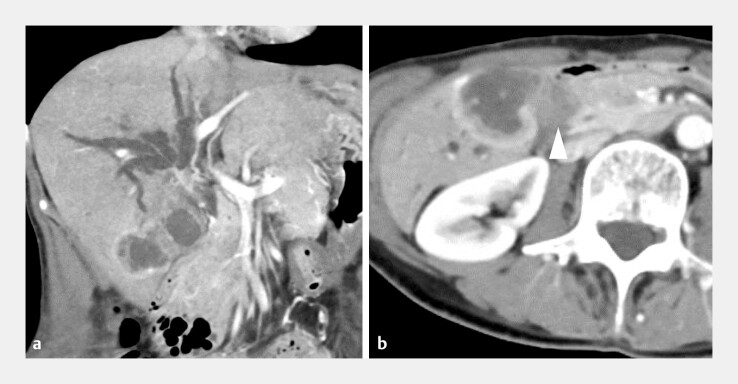
Computed tomographic images before biliary drainage. **a** Coronal imaging shows a bismuth type IIIa hilar biliary obstruction caused by advanced gallbladder cancer. **b** The duodenum is obstructed by tumor invasion.

**Fig. 3:**
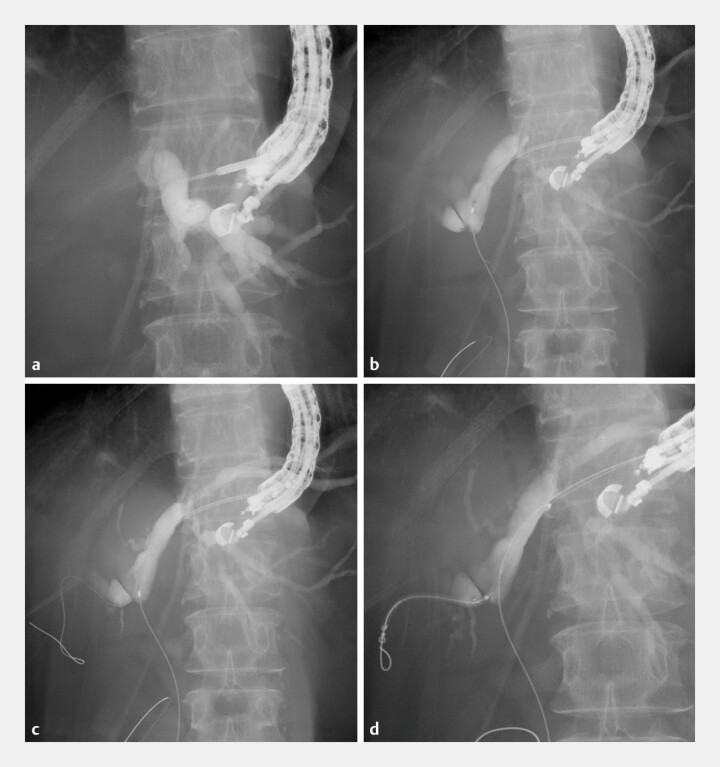
Endoscopic ultrasonography-guided hepaticogastrostomy. **a** The intrahepatic bile duct is punctured using a 19-gauge needle. **b** A guidewire is advanced across the stricture, and cholangiography confirms hilar biliary obstruction. **c** A second guidewire is inserted into the right anterior branch using a double-lumen catheter. **d** The slim delivery system of the first stent is smoothly advanced into the right anterior branch.

**Fig. 4:**
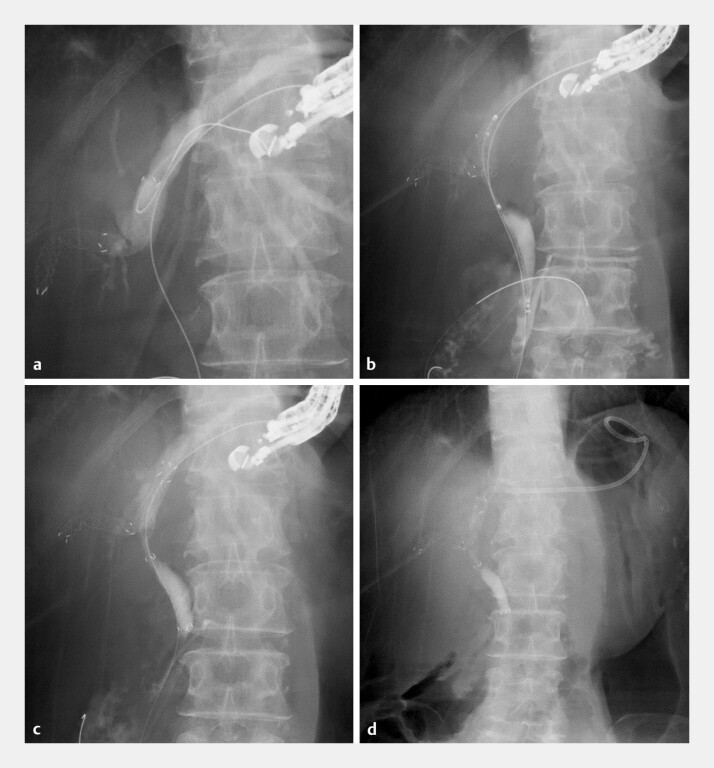
Bridging and antegrade stent-in-stent placement under EUS-HGS guidance. **a** After deployment of the first multi-hole metal stent as a bridging stenting, the guidewire is manipulated through a side hole toward the common bile duct. **b** The guidewire is advanced across the stricture, followed by 6-mm balloon dilation of both the side hole and the stricture. **c** The second multi-hole metal stent is deployed as an antegrade stent through the side hole of the first stent. **d** A 7-Fr plastic stent is finally placed through the hepaticogastrostomy tract. EUS-HGS, endoscopic ultrasound-guided hepaticogastrostomy.

Endoscopic ultrasound-guided hepaticogastrostomy (EUS-HGS) has been used in combination with bridging and antegrade stenting 1 2 ; however, uncovered metal stents often suffer from tumor ingrowth and limited patency 3 . A newly developed multi-hole metal stent with a 5.9-Fr slim delivery system (HANAROSTENT Biliary Multi Hole Benefit; M.I. Tech Co., Ltd, Pyeongtaek, South Korea) has been developed ( Fig. 1 4 5 ). Herein, we report a novel EUS-HGS technique achieving simultaneous bridging and antegrade stent-in-stent placement using multi-hole metal stents ( Video 1 ). A 67-year-old woman was referred to our hospital with hilar biliary obstruction due to advanced gallbladder cancer ( Fig. 2 ). Because the duodenum was obstructed, EUS-HGS was selected as the initial drainage approach ( Fig. 3 , Fig. 4 ). The intrahepatic bile duct was punctured using a 19-gauge needle, and after contrast injection, a 0.025-inch guidewire was advanced across the stricture into the common bile duct, followed by insertion of a double-lumen catheter. Cholangiography revealed a bismuth type IIIa stricture, and a second guidewire was inserted into the right anterior branch. A slim delivery system for the multi-hole metal stent was smoothly advanced across the hilar stricture into the anterior brunch. The first multi-hole metal stent (6 mm and 6 cm) was deployed as a bridging stent from the right to the left intrahepatic bile duct. The guidewire was then manipulated through a side hole of the first stent toward the common bile duct, and both the side hole and the stricture were dilated using a balloon catheter. A second multi-hole metal stent was inserted as an antegrade stent through the side hole, resulting in successful partial stent-in-stent placement. Finally, a plastic stent was placed through the hepaticogastrostomy tract. To the best of our knowledge, this is the first report of EUS-HGS with simultaneous bridging and antegrade stent-in-stent placement using multi-hole metal stents. This technique may offer prolonged patency for hilar biliary obstruction with duodenal stenosis.

A novel EUS-HGS technique achieving simultaneous bridging and antegrade stent-in-stent placement using multi-hole metal stents. EUS-HGS, endoscopic ultrasound-guided hepaticogastrostomy.Video 1

Endoscopy_UCTN_Code_TTT_1AS_2AH
